# International operations and corporate R&D investment: Evidence from China

**DOI:** 10.1371/journal.pone.0308290

**Published:** 2024-09-10

**Authors:** Ying Fu, Jiaying Li, Ying Wu

**Affiliations:** 1 School of Finance, Southwestern University of Finance and Economics Wenjiang, Chengdu, Sichuan; 2 School of Business, Nanjing Normal University, Xianlin University City, Nanjing, Jiangsu Province, China; An-Najah National University, STATE OF PALESTINE

## Abstract

This paper presents an examination of the relationship between international operations and corporate R&D investment. Using a large sample of Chinese listed firms for the 2009–2022 period and the ordinary least squares method, we find that international operations have a positive effect on corporate R&D investment. The finding remains valid after a battery of robustness tests. Mechanism tests show that international operations increase corporate R&D investment by diversifying product demand instead of increasing firms’ international knowledge acquisition. This paper provides new evidence on the role of international operations in innovation activities.

## 1. Introduction

With the deepening of globalization, more firms have engaged in international operations [1–5]. Previous studies have paid more attention to the effect of international operations on firms’ operations in developed economies [6]. However, firms from developing countries also actively participate in international operations. Emerging market multinational enterprises lack advanced technologies and established brands and have different business motives [7]. Examining the economic results of multinational firms from emerging countries can improve our understanding of increasing international business trends.

Innovation is a crucial driver of firm competitiveness and has attracted considerable attention from the academic community [8]. Although existing studies test the relationship between international operations and corporate innovation outputs, scholars fail to obtain a consistent view. For example, many scholars find that international operations increase corporate innovations [7, 9, 10]. However, Zheng et al [11] found a nonlinear association between international operations and innovation outputs. These inconsistent findings may be due to the sample or model selection. In this paper, we examine the effect of international operations on corporate R&D investment. This is because innovation is a risky, long-term, and idiosyncratic process [12]. Firms may fail to obtain innovation outputs after investing resources in R&D projects. Therefore, R&D investment can better reflect corporate engagement in innovation activities.

International operations may increase corporate R&D investment. First, international operations expand the product market, which needs firms to develop new products to meet overseas customers’ needs, thus inducing firms to increase R&D investment. In addition, multinational firms from emerging markets usually tend to develop international business in developed economies with cutting-edge technologies, which helps them to improve their knowledge stock and learn advanced technological content [7, 13], thus increasing their incentive to expand R&D investment to absorb knowledge and develop new technologies.

We test the effect of international operations on corporate R&D investment by using data from listed firms in China. Chinese data are beneficial for this study for two reasons. First, China, as the largest developing country, has actively encouraged domestic enterprises to participate in internationalization. For example, one of the objectives of the “Belt and Road” policy issued by the government is to encourage more Chinese firms to engage in international operations(China issued the “Belt and Road” policy to achieve economic development in 2013. The Chinese government has signed more than 200 documents of cooperation with 152 countries and 32 international organizations to jointly build the “Belt and Road”.). According to the “World Investment Report 2023” of the United Nations Conference on Trade and Development, China’s outward foreign direct investment flow ranks second in the world. Second, China is also the largest country that is engaging in innovation. According to the latest data from the World Intellectual Property Indicators Report in 2022, the number of patent applications in China was approximately 1.58 million, which was nearly half of all global patent applications. Therefore, China provides an appropriate setting for this empirical study.

Following previous studies [14–16], we use the ratio of R&D expenditures to total assets to measure R&D investment. In addition, following Liu et al [17], we use a dummy variable to indicate whether a firm engages in international operations in a year. Using Chinese A-share listed firms for 2009 to 2022, we find that international operations have a positive effect on R&D investment. The finding remains valid after changing the measurement of R&D activities, controlling for the effect of audit quality, excluding the impact of the “Belt and Road” policy, and replacing the identification method.

The following methods are then used to build a valid causal relationship between international operations and R&D investment. First, to alleviate endogeneity problems from selection bias, we use the propensity score matching (PSM) method to enable firms’ characteristics to be comparable between firms with and without international operations. Next, we examine the relationship between international operations and R&D investment. Second, we use the mean value of international operations in the province where a firm is located as the instrumental variable and the two-stage instrumental variable method to examine the effect of international operations on corporate R&D investment. The empirical results suggest that international operations increase corporate R&D investment.

Finally, we also examine the mechanisms through which international operations impact corporate R&D investment. We produce suggestive evidence on how international operations affect corporate R&D investment because these channels are not easily observable. First, compared with firms in industries with less production market competition, firms facing fierce production market competition are incentivized to invest more in R&D to develop more new productions to cater to foreign customers, obtaining larger overseas market shares. Therefore, we can expect that the relationship between international operations and corporate R&D investment is prominent for firms with higher production market competition when international operations affect corporate R&D investment by diversifying product demand. In addition, high-tech firms have more advanced technologies and less demand to learn and absorb knowledge from overseas. Thus, we can expect that the effect of international operations on corporate R&D investment is weaker when international operations increase R&D investment by accessing overseas knowledge. The empirical results show that the impact of international operations on corporate R&D investment is prominent for firms with fierce production market competition, but we fail to find a significant difference in the relationship between international operations and corporate R&D investment in high-tech firms and nonhigh-tech firms. These results indicate that international operations affect corporate R&D investment by diversifying product demand.

This paper contributes to the literature in the following ways. First, we provide new evidence on whether Chinese firms engaging in international operations can absorb external knowledge. Previous studies indicated that firms with outward foreign direct investment can increase innovation outputs by accessing advanced overseas knowledge [7, 13]. However, the findings of this paper do not support the view that international operations help firms absorb more advanced knowledge.

In addition, the findings of this paper support the view that international operations are conducive to innovation activities. Although many papers show a positive effect of international operations on innovation activities [7, 9, 10, 13, 18], Zheng et al [11] found that there is a curvilinear relationship between Chinese firms’ outward foreign direct investment and radical innovation performance. The positive effect of international operations on corporate R&D investment and innovation outputs in this paper suggests that international operations improve firms’ innovative abilities.

Finally, this paper enriches the literature on the factors affecting corporate R&D investment. The existing papers have examined how firms’ internal and external factors affect R&D investment. With respect to internal factors, scholars have noted that corporate governance [19, 20], executive characteristics [21–23], and financial characteristics [24, 25] play crucial roles in corporate R&D investment. In addition, external factors, such as government regulation [26–28], religion [29], and social trust [30], also impact corporate R&D investment. The findings of this paper suggest that international operations are a key factor in stimulating more corporate R&D investment.

The rest of the paper is organized as follows. Section 2 develops the hypothesis. Section 3 introduces the research design. Section 4 presents the empirical results. Section 5 presents the conclusions.

## 2. Hypothesis development

International operations may increase corporate R&D investment through the following two channels. First, international investment may increase corporate R&D investment by diversifying production demand. Entering a new foreign market means that firms must face customers with different cultural backgrounds and preferences. There is a high possibility that existing products, which are designed on the basis of domestic consumer preferences, fail to meet foreign consumer needs. Firms need to perform production innovation. The process requires firms to invest more resources in R&D activities [31]. Second, international operations may increase corporate R&D investment by increasing firms’ international knowledge acquisition. Multinational firms from emerging countries are usually technology catchers, and they can learn and accumulate external knowledge from the internationalization process [7, 13]. To apply overseas knowledge to break technological bottlenecks, firms must invest resources to absorb and develop new knowledge. Therefore, international operations may have a positive effect on corporate R&D investment.

Hypothesis: International operations increase corporate R&D investment.

## 3. Research design

### 3.1 Sample

To construct our sample, we start with all Chinese A-share public firms from 2009 to 2022. The sample period begins in 2009 because we want to exclude the effect of the global financial crisis in 2008. Firm data are collected from the China Securities Market and Accounting Research (CSMAR) and Chinese Research Data Service (CNRDS) databases. The databases provide detailed Chinese listed firms’ financial data and macroeconomic data. They are common and important databases used in Chinese research [32–35]. Then, we exclude financial services firms and firm-year observations with missing information for the variables. Our final sample includes 36224 firm-year observations representing 4983 individual firms. To alleviate the impact of outliers, we also winsorize all continuous variables at the 1% and 99% levels.

### 3.2 The measurement of international operations

Following Liu et al [17], we use a dummy variable to indicate whether firms engage in international operations (International). Specifically, International equals 1 when a firm builds branches outside the Chinese mainland and 0 otherwise.Please note that we regard firms that build branches in Hong Kong, Macao, and Taiwan as international operations participators. This is because although these three regions belong to China, they have different economic and political systems from that of the Chinese mainland.

### 3.3 The measurement of corporate R&D investment

Following previous studies [14, 15], we use the ratio of R&D expenditures to total assets to measure corporate R&D investment (RD). A larger RD means more R&D investment.

### 3.4 Empirical methodology

Following previous studies [24, 36], we use the following method to study the relationship between international operations and corporate R&D investment.

$${RD}_{i,t}=\beta_{0}+\beta_{1}{International}_{i,t}+\gamma^{'}{Control}_{i,t}+\eta_{j}+\mu_{t}+\varepsilon_{i,t}$$ (1)

where i denotes an individual firm and t denotes the year. The control variables (Control) are potential factors that have been shown in previous studies to predict corporate R&D investment [14, 15, 22, 24, 28, 37]. Specifically, we use the natural logarithm of the book value of assets to control for the effect of firm size (Size)(Please note the monetary unit of Size is Yuan). We use the ratio of tangible assets to total assets to account for the effect of capital intensity (Tangi). Lev (i.e., the ratio of total debts to total assets) and the operating cash flow scaled by total assets (Cash) are added to control for the impact of capital structure. Return on assets (ROA), which equals net profits scaled by total assets, is used to control for the effect of profitability. We use the Herfindahl index, which equals the sum of squared market shares in the sales of a firm’s industry, to control for the effect of product market competition. The index is used to measure product market competition. A large value of the index means lower product market competition. The natural logarithm of firm age (Age) is used to capture the impact of a firm’s life cycle. In addition, we include the ratio of independent directors to total directors to capture the impact of corporate governance (Indep). To control for the effect of CEO power [38], we use a dummy variable to indicate whether the CEO and chairperson are the same person (Duality). Ownership plays a crucial role in firms’ operations in China [39]. We include a dummy variable to indicate whether a firm is a state-owned enterprise (SOE). Finally, we also include industry fixed effects (η) to control for unobservable time-invariant industry-specific characteristics and year fixed effects (μ) to control for common time trends. All the variables are defined in Table 1.

**Table 1:** Variable definition.

Variable	Definition
RD	The ratio of R&D expenditures to total assets
International	A dummy variable that equals 1 when a firm builds branches outside the Chinese mainland and 0 otherwise
Size	The natural logarithm of the book value of assets
Lev	The ratio of total debts to total assets
Cash	The operating cash flow scaled by total assets
Tangi	The ratio of tangible assets to total assets
ROA	Net profit scaled by total assets
HHI	The sum of squared market shares in the sales of a firm’s industry
Age	The natural logarithm of firm age
Indep	The ratio of independent directors to total directors
Duality	A dummy variable that equals 1 when the CEO and the chairperson are the same person and 0 otherwise
SOE	A dummy variable that equals 1 when a firm is a state-owned enterprise and 0 otherwise

### 3.5 Descriptive statistics

Panel A of Table 2 reports the descriptive statistics of the variables. The mean and maximum values of RD are 0.0234 and 0.1150, respectively, suggesting that there is greater heterogeneity in corporate R&D investment. In addition, the mean value of International suggests that 51.40% of observations engage in international operations. This also reflects that Chinese firms are the key participants in international investment [7, 40]. The mean value of Size is 22.0625, which is greater than that in Ghana [41]. In addition, the mean value of Lev is 0.4070, which is greater than that in the U.S. [42]; this is consistent with the fact that the equity market’s underdevelopment makes enterprises rely heavily on debt as external financing. Finally, the mean value of Indep is 0.3832, indicating that independent directors account for 38.32% of all directors.

**Table 2:** Descriptive statistics. Panel A displays the descriptive statistics used in the main regression. Panel B presents the mean value of RD investment and international operations among industries. The sample period is 2009–2022.

Panel A									
Variable	N	Mean	Std		Minimum		Median		Maximum
RD	36224	0.0234	0.0209		0.0001		0.0194		0.1150
International	36224	0.5140	0.4998		0.0000		1.0000		1.0000
Size	36224	22.0625	1.2863		19.7397		21.8618		26.1532
Lev	36224	0.4070	0.2078		0.0487		0.3944		0.9343
Cash	36224	0.0466	0.0693		-0.1579		0.0454		0.2487
Tangi	36224	0.2013	0.1451		0.0034		0.1720		0.6493
ROA	36224	0.0375	0.0719		-0.3174		0.0404		0.2112
HHI	36224	0.0482	0.0782		0.0074		0.0153		0.3884
Age	36224	2.8714	0.3536		1.7918		2.9444		3.5264
Indep	36224	0.3832	0.0727		0.2500		0.3684		0.6000
Duality	36224	0.3240	0.4680		0.0000		0.0000		1.0000
SOE	36224	0.2926	0.4550		0.0000		0.0000		1.0000
Panel B									
Industry				N		RD		International	
Agriculture				447		0.0076		0.2908	
Mining				733		0.0086		0.5020	
Food and textile manufacturing				2423		0.0123		0.4808	
Chemical manufacturing				7644		0.0223		0.4432	
Metal and equipment manufacturing				15800		0.0269		0.5527	
Other manufacturing				865		0.0273		0.5110	
Electricity, gas, and water				694		0.0025		0.3386	
Building and construction				895		0.0137		0.5318	
Commerce				877		0.0073		0.6032	
Transportation and logistics				556		0.0041		0.6475	
Accommodation and restaurant				46		0.0027		0.2826	
Information technology				2918		0.0469		0.5517	
Real estate				406		0.0025		0.5394	
Leasing and commercial services				345		0.0094		0.6232	
Research and technical services				518		0.0258		0.4402	
Environment and public facilities management				403		0.0112		0.4045	
Residential services, repair, and other services				14		0.0214		0.5000	
Education				42		0.0232		0.3095	
Health and social work				78		0.0148		0.7308	
Culture, sport, and entertainment				334		0.0093		0.5269	
Conglomerates				186		0.0087		0.5000	
Total				36224		0.0234		0.5140	

We also report the mean value of RD investment and international operations across industries in Panel B of Table 2 (Following Li et al [43], we classify the firms in the manufacturing industry into more detailed industry categories using the 2-digit code.). In terms of corporate R&D investment, firms in the information technology industry have the highest R&D investment. This is consistent with the fact that the information technology industry is the main source of technological advancement worldwide. Finally, we find that the accommodation and restaurant industry has fewer international operations.

### 3.6 Correlation matrix

Table 3 reports the correlation coefficient matrix. We find that the correlation coefficient between international operations and corporate R&D investment is positive and significant, suggesting that international investment is positively related to corporate R&D investment. In addition, the correlation coefficients between international operations and the control variables are low, suggesting that multicollinearity is not a serious problem. Finally, the correlation between firm age and R&D investment is similar to the findings of Chen et al [44].

**Table 3:** Correlation matrix.

	RD	International	Size	Lev	Cash	Tangi	ROA	HHI	Age	Indep	Duality	SOE
RD	1											
International	0.137***	1										
Size	-0.202***	0.276***	1									
Lev	-0.211***	0.104***	0.476***	1								
Cash	0.060***	0.038***	0.072***	-0.161***	1							
Tangi	-0.201***	-0.088***	0.129***	0.148***	0.201***	1						
ROA	0.103***	-0.028***	-0.024***	-0.388***	0.406***	-0.071***	1					
HHI	0.034***	-0.021***	0.024***	0.005	-0.021***	-0.162***	-0.019***	1				
Age	-0.057***	0.075***	0.218***	0.178***	0.024***	0.031***	-0.119***	-0.083***	1			
Indep	0.050***	0.056***	-0.043***	-0.056***	0.012**	-0.041***	0.023***	0.007	-0.027***	1		
Duality	0.129***	0.047***	-0.187***	-0.152***	-0.010*	-0.102***	0.042***	-0.018***	-0.094***	0.096***	1	
SOE	-0.194***	-0.113***	0.369***	0.305***	-0.007	0.185***	-0.085***	0.056***	0.161***	-0.149***	-0.307***	1

This table displays the pairwise correlation coefficients between international operations, R&D investment, and the control variables. The sample period is 2009–2022. The symbols

*, **, and *** denote significance at the 10%, 5%, and 1% levels, respectively.

## 4 Empirical results

### 4.1 Main results

Table 4 reports the regression results of Model 1. We only regress R&D investment on international operations in Column 1 of Table 4. The coefficient of International is positive and significant at the 1% level, suggesting that international operations have a positive effect on corporate R&D investment. In Column 2, we further control for firm size, leverage, operating cash flow, tangible assets, return on assets, and product market competition. The coefficient of International is positive and significant. In the last column, we include all control variables. The coefficient of International is positive and significant at the 1% level, which supports the hypothesis that international operations increase corporate R&D investment.

**Table 4:** The effect of international operations on corporate R&D investment.

	(1)	(2)	(3)
	RD	RD	RD
International	0.0038***	0.0052***	0.0051***
	(0.0002)	(0.0002)	(0.0002)
Size		-0.0023***	-0.0022***
		(0.0001)	(0.0001)
Lev		-0.0029***	-0.0020***
		(0.0006)	(0.0006)
Cash		0.0198***	0.0204***
		(0.0017)	(0.0017)
Tangi		-0.0128***	-0.0126***
		(0.0007)	(0.0007)
ROA		0.0206***	0.0196***
		(0.0021)	(0.0021)
HHI		0.0011	0.0005
		(0.0038)	(0.0037)
Age			-0.0045***
			(0.0003)
Duality			0.0011***
			(0.0002)
Indep			0.0009
			(0.0013)
SOE			0.0007***
			(0.0002)
Constant	-0.0010	0.0508***	0.0587***
	(0.0008)	(0.0021)	(0.0024)
Industry fixed effects	Yes	Yes	Yes
Year fixed effects	Yes	Yes	Yes
N	36224	36224	36224
R^2^	0.2729	0.3146	0.3194

The table reports the regression results of Model 1. International is a dummy variable. RD is the ratio of R&D expenditures to total assets. The sample period is 2009–2022. The standard errors are shown in parentheses. The symbols

*, **, and *** denote significance at the 10%, 5%, and 1% levels, respectively.

In terms of the control variables, the coefficient of Size is negative, meaning that larger firms invest proportionally less in R&D activities, which is consistent with the findings of Zhang et al [45]. In addition, higher leverage (Lev) reduces corporate R&D investment because higher leverage lowers firms’ risk-bearing levels for R&D projects. Finally, the coefficient of SOE is positive and significant, suggesting that state-owned enterprises tend to invest more in R&D activities than nonstate-owned enterprises do [46].

### 4. 2 Robustness tests

In this subsection, we run four robustness tests. First, we change the measurement of corporate R&D investment. Following previous studies [20, 28, 36], we use R&D expenses divided by sales to measure corporate R&D investment (RD1). As shown in Column 1 of Table 5, the coefficient of International is positive and significant, suggesting that international operations stimulate corporate R&D investment.

**Table 5:** Robustness tests.

	(1)	(2)	(3)	(4)	(5)
	RD1	Innovation	RD	RD	RD
International	0.0055***	0.1754***	0.0050***	0.0054***	0.0011***
	(0.0005)	(0.0145)	(0.0002)	(0.0002)	(0.0002)
Big10			0.0019***		
			(0.0002)		
Constant	0.0998***	-10.2615***	0.0543***	0.0645***	0.0975***
	(0.0054)	(0.1882)	(0.0024)	(0.0028)	(0.0035)
Controls	Yes	Yes	Yes	Yes	Yes
Industry fixed effects	Yes	Yes	Yes	Yes	No
Year fixed effects	Yes	Yes	Yes	Yes	Yes
Firm fixed effects	No	No	No	No	Yes
N	36220	28360	35374	28800	36224
R^2^	0.3614	0.4279	0.3212	0.3154	0.0980

The table reports the results of the robustness tests. In Column 1, we change the measurement of R&D investment. We also examine the effect of international operations on innovation outputs in Column 2. In Column 3, we control for the effect of audit quality. We exclude the effect of the “Belt and Road” Initiative on R&D investment in Column 4. In the last column, we control for firm fixed effects instead of industry fixed effects. The sample period is 2009–2022. The standard errors are shown in parentheses. The symbols

*, **, and *** denote significance at the 10%, 5%, and 1% levels, respectively. Please note that the control variables are included in the regression specifications but the coefficient estimates are not reported to preserve space.

Then, R&D investment reflects innovation inputs instead of innovation outputs. To fully understand the role of international operations in corporate innovation activities, we also examine the effect of international operations on innovation outputs. Specifically, following Kong et al [47], we use the natural logarithm of the total number of invention applications that are eventually granted and utility patent applications that are eventually granted to measure innovation outputs (Innovation). In addition, considering that the innovation process is a long-term process, we use innovation outputs in the t+1 period as the dependent variable. As shown in Column 2 of Table 5, the coefficient of International suggests that international operations increase corporate innovation outputs.

In addition, we control for the effect of audit quality. The existing papers fail to obtain a consistent view of the role of auditors in innovation activities [48, 49]. In Column 3 of Table 5, we use a dummy variable to indicate whether a firm has a high-quality audit (Big10). Big10 equals 1 when a firm is audited by the audit company ranked in the top 10 and 0 otherwise. The empirical results show that international operations have a positive effect on corporate R&D investment, even if we consider the effect of audit quality. Moreover, the coefficient of Big10 is positive and significant, suggesting that higher audit quality increases R&D investment. This is consistent with the findings of Wang et al [49].

In addition, we consider the policy shock of the “Belt and Road” in 2013. Previous studies have shown that the “Belt and Road” policy affects corporate innovation activities, such as innovation inputs [50] and innovation outputs [31]. To exclude the effect of the policy, we use only observations after 2013 to examine the relationship between international operations and corporate R&D investment. As shown in Column 4 of Table 5, the coefficient of International is positive and significant at the 1% level, meaning that international operations are conducive to R&D investment.

Finally, although we attempt to control for more firm characteristics that may affect firms’ R&D investment decisions, some firm characteristics that cannot be measured can impact R&D expenses. Therefore, we change the identification method. Specifically, we control for firms’ fixed effects in our analysis. As shown in Column 5 of Table 5, the coefficient of International is positive and significant at the 1% level. The findings are consistent with those in Column 3 of Table 4.

### 4.3 Endogeneity corrections

The positive relationship between international operations and corporate R&D investment may be affected by endogeneity problems. Choosing to participate in international operations may not be random. Many factors determine a firm’s choice of international operations to the extent that these factors may be correlated with R&D investment. To mitigate potential endogeneity concerns, we run the following methods.

First, to make the characteristics of firms comparable between firms with and without international operations, we use the propensity score matching (PSM) method to match our sample. Specifically, following Kong et al [47], we use one-to-one nearest neighbor matching of propensity scores with replacement and a caliper of 0.01, which are estimated by the logistic regression of International on a set of firm characteristics. Please note that we include industry fixed and year fixed effects in the matching regression. Appendix A displays the balanced test results, which consist of pairwise comparisons of the covariates on which the matching is performed after and before the matching process. We find that there are no statistically significant differences across the overall characteristics of firms after PSM. Panel A of Table 6 reports the regression results when the matched samples are used. The coefficient of International is positive and significant at the 1% level, indicating that international operations increase corporate R&D investment.

**Table 6:** Endogeneity corrections.

Panel A			
		(1)	
		RD	
International		0.0053***	
		(0.0003)	
Constant		0.0550***	
		(0.0036)	
Controls		Yes	
Industry fixed effects		Yes	
Year fixed effects		Yes	
N		17128	
R^2^		0.3176	
Panel B			
	(2)		(3)
	International		RD
International			0.0191***
			(0.0008)
IVinternational	4.7015***		
	(0.1066)		
Constant	-18.8923***		0.0991***
	(0.3554)		(0.0035)
Controls	Yes		Yes
Industry fixed effects	Yes		Yes
Year fixed effects	Yes		Yes
N	36224		36224
R^2^	0.1795		0.3180

The table displays the results of the endogeneity corrections. Panel A reports the results of the PSM method. Panel B reports the results of the two-stage instrumental variable method. The sample period is 2009–2022. The standard errors are shown in parentheses. The symbols

*, **, and *** denote significance at the 10%, 5%, and 1% levels, respectively. Please note that the control variables are included in the regression specifications but the coefficient estimates are not reported to preserve space.

Second, we also use the two-stage instrumental variable method to examine the relationship between international operations and corporate R&D investment. Specifically, we use the average value of international operations in a province where a firm is located (IVinternational). On the one hand, the average value of international operations in a province is related to a firm’s overseas business strategy. On the other hand, the average value of international operations in a province does not directly affect a firm’s R&D investment decisions. In Column 1 of Panel B, following Mobbs et al [51], we use the logistic regression model to examine the relationship between international operations (International) and the instrumental variable (IVinternational) because the dependent variable in the first stage of the two-stage instrumental variable method is a dummy variable. The coefficient of IVinternational is positive and significant at the 1% level, suggesting that the instrumental variable is efficient and valid. Column 2 of Panel B of Table 6 also represents the second-stage results of the two-stage instrumental variable method. The coefficient of International is positive and significant, meaning that international operations increase corporate R&D investment.

### 4.4 Mechanism tests

Obviously, we cannot directly measure overseas consumers’ demand preferences or whether firms learn advanced knowledge from the overseas market. Therefore, our goal in this subsection is to provide suggestive evidence that could advance our understanding of the mechanisms through which international operations affect corporate R&D investment.

First, although entering a new market requires firms to invest resources in developing new products to satisfy overseas consumers’ demand preferences, firms facing different product market competition stresses have heterogeneous incentives to invest in R&D projects. Fierce product market competition lowers firms’ current product attractiveness [28]. This means that to obtain more market share and survive overseas, firms in industries with intense product market competition may invest more in R&D activities to develop more new products to cater to overseas consumers. Therefore, we expect the positive relationship between international operations and corporate R&D investment to be attenuated for firms in concentrated industries.

Second, high-tech firms have more advanced technologies and greater absorptive abilities than other firms do. Even if multinational firms from emerging markets learn advanced knowledge from the overseas market, we can expect that high-tech firms invest few resources to absorb external knowledge and use overseas knowledge in innovation activities with lower costs. Therefore, the effect of international operations on corporate innovation is attenuated for high-tech firms when international operations affect corporate R&D investment by accessing external advanced knowledge.

Following previous studies [52, 53], we use the following method to examine these mechanisms.

$$\begin{array}{l} {RD}_{i,t}=\alpha_{1}+\alpha_{1}{International}_{i,t}\times {HHI}_{i,t}+\alpha_{2}{International}_{i,t}\\ \;+{\alpha_{3}{HHI}_{i,t}+\gamma }^{'}{Control}_{i,t}+\eta_{j}+\mu_{t}+\varepsilon_{i,t} \end{array}$$ (2)

$$\begin{array}{l} {RD}_{i,t}=\kappa_{1}+\kappa_{1}{International}_{i,t}\times {High}_{i,t}+\kappa_{2}{High}_{i,t}\\ \;+\kappa_{3}{International}_{i,t}+\gamma^{'}{Control}_{i,t}+\eta_{j}+\mu_{t}+\varepsilon_{i,t} \end{array}$$ (3)

where i denotes individual firms and t denotes the year. High is a dummy variable that equals 1 when listed firms or their subsidiaries obtain high-tech qualification recognition from the government and 0 otherwise.

As shown in Column 1 of Table 7, the coefficient of International×HHI is negative and significant at the 1% level, suggesting that the effect of international operations on corporate R&D investment is attenuated for firms in industries with weaker product market competition. Moreover, the findings also indicate that international operations increase corporate R&D investment by diversifying production demand.

**Table 7:** Mechanism tests.

	(1)	(2)
	RD	RD
International×HHI	-0.0104***	
	(0.0029)	
International	0.0056***	0.0049***
	(0.0002)	(0.0003)
International×High		-0.0002
		(0.0004)
High		0.0042***
		(0.0002)
Constant	0.0586***	0.0628***
	(0.0024)	(0.0024)
Controls	Yes	Yes
Industry fixed effects	Yes	Yes
Year fixed effects	Yes	Yes
N	36224	36224
R^2^	0.3197	0.3277

The tables report empirical results on how international operations affect corporate R&D investment. The sample period is 2009–2022. The standard errors are shown in parentheses. The symbols

*, **, and *** denote significance at the 10%, 5%, and 1% levels, respectively. Please note that the control variables are included in the regression specifications but the coefficient estimates are not reported to preserve space.

In addition, we find that the coefficient of International×High is insignificant in Column 2, suggesting that there is no difference in the relationship between international operations and corporate R&D investment for high-tech and nonhigh-tech firms. This finding means that international operations fail to affect corporate innovation by increasing firms’ international knowledge acquisition. This may be because, compared with other emerging countries, China has significantly reduced the technological gap with developed countries in recent years. In addition, developed countries also try to maintain technological advantages by limiting technological flow to the country. For example, the U.S. issued a technology war and set a technology blockade against China in recent years. Finally, tacit knowledge transmission needs spatial proximity. Establishing overseas R&D centers is a common way to access local innovation processes. However, few firms built overseas R&D centers during the sample period.

## 5 Conclusion

International operations have been a crucial corporate strategy in emerging markets. In this paper, we examine the effect of international operations on corporate R&D investment. Using Chinese A-share listed firms from 2009 to 2022, we find that international operations have a positive effect on corporate R&D investment. The finding remains valid after changing the measurement of R&D activities, controlling for the effect of audit quality, excluding the effect of the “Belt and Road” policy, and replacing the identification method. Mechanism tests show that international operations increase corporate R&D investment by diversifying product demand instead of increasing firms’ international knowledge acquisition. The findings of this paper provide new and different evidence on how international operations influence corporate innovation activities.

The findings of this paper have significant implications for multinational firms and policy-makers. First, multinational firms from emerging markets should expand their operations in the international market, which helps them increase innovation inputs. In addition, they should establish research centers overseas to absorb international advanced knowledge instead of developing R&D activities mainly in-house. With respect to policy-makers, leaders should actively embrace globalization instead of trade protection to improve corporate competitiveness.

There are several limitations to this study. First, the findings of this paper explain only limited channels through which international operations affect corporate R&D investment. In addition, more evidence on the relationship between international operations and corporate investment needs to be provided for emerging economies. Finally, this paper uses only listed firms to examine the effect of international operations on corporate R&D investment. However, listed firms account for only a smaller portion of all firms around the world; thus, scholars need to collect and test whether international operations increase R&D investment in unlisted firms.

## Supporting information

S1 Appendix (DOCX)
