# Supplementary material for: International operations and corporate R&D investment: Evidence from China
Source: PLoS One. 2024 Sep 10;19(9):e0308290. doi: 10.1371/journal.pone.0308290 (PMC11386441; doi:10.1371/journal.pone.0308290)
Supplement: S1 Appendix — (DOCX) [file pone.0308290.s001.docx]

Appendix A

| Variable | Sample | International=1 | International=0 | % bias | bias | t | p |
| --- | --- | --- | --- | --- | --- | --- | --- |
| Size | U | 22.407 | 21.698 | 57.5 |  | 54.61 | 0.000 |
|  | M | 22.405 | 22.423 | -1.4 | 97.5 | -1.27 | 0.205 |
| Lev | U | 0.42804 | 0.38476 | 20.9 |  | 19.93 | 0.000 |
|  | M | 0.42792 | 0.43366 | -2.8 | 86.7 | -2.63 | 0.009 |
| Cash | U | 0.0492 | 0.04386 | 7.7 |  | 7.33 | 0.000 |
|  | M | 0.04919 | 0.04996 | -1.1 | 85.5 | -1.07 | 0.283 |
| Tangi | U | 0.18898 | 0.21442 | -17.6 |  | -16.74 | 0.000 |
|  | M | 0.18905 | 0.18883 | 0.1 | 99.1 | 0.15 | 0.883 |
| ROA | U | 0.03553 | 0.03949 | -5.5 |  | -5.24 | 0.000 |
|  | M | 0.03551 | 0.03439 | 1.6 | 71.6 | 1.45 | 0.148 |
| HHI | U | 0.04659 | 0.0498 | -4.1 |  | -3.90 | 0.000 |
|  | M | 0.04662 | 0.04765 | -1.3 | 67.9 | -1.32 | 0.186 |
| Age | U | 2.8973 | 2.8441 | 15.1 |  | 14.36 | 0.000 |
|  | M | 2.8977 | 2.9015 | -1.1 | 92.8 | -1.07 | 0.286 |
